# Changes in Homelessness Among US Veterans After Implementation of the Ending Veteran Homelessness Initiative

**DOI:** 10.1001/jamanetworkopen.2023.53778

**Published:** 2024-01-29

**Authors:** Thomas P. O’Toole, Lisa M. Pape, Vincent Kane, Monica Diaz, Anne Dunn, James L. Rudolph, Shereef Elnahal

**Affiliations:** 1 US Veterans Health Administration, Washington, DC; 2 Providence Veterans Affairs Health System Center for Innovation in Long Term Services and Support, Providence, Rhode Island; 3 Division of Medicine and Biology, Alpert Medical School at Brown University, Providence, Rhode Island

## Abstract

**Importance:**

Homelessness is a persistent and growing problem. What role health systems should play and how that role is incorporated into larger strategic efforts are not well-defined.

**Objective:**

To compare homelessness among veterans with that in the general population during a 16-year study period before and after implementation of the Ending Veteran Homelessness Initiative, a program to rehouse veterans experiencing homelessness.

**Design, Setting, and Participants:**

This national retrospective cohort study using a mixed-methods approach examined annualized administrative (January 1, 2007, to December 31, 2022) and population data prior to (2007-2009) and during (2010-2022) the Ending Veteran Homelessness initiative. Participants included unhoused adults in the US between 2007 and 2022.

**Exposure:**

Enrollment in Veterans Health Administration (VHA) Homeless Program Office components providing housing, case management, and wrap-around clinical and supportive services.

**Main Outcomes and Measures:**

Point-in-time (PIT) count data for unhoused veterans and nonveterans during the study period, number of Section 8 housing vouchers provided by Housing and Urban Development–Veterans Administration Supportive Housing, number of community grants awarded by Supportive Services for Veterans and Families, and total number of veterans housed each year. Semistructured interviews with VHA leadership were performed to gain insight into the strategy.

**Results:**

In 2022, 33 129 veterans were identified in the PIT count. They were predominantly male (88.7%), and 40.9% were unsheltered. During the active years of the Ending Veteran Homelessness initiative, veteran homelessness decreased 55.3% compared with 8.6% for the general population. The proportion of veterans in this cohort also declined from 11.6% to 5.3%. This change occurred during a shift to “housing first” as agency policy to create low-barrier housing availability. It was also coupled with substantial growth in housing vouchers, grants to community partner agencies, and growth in VHA clinical and social programming to provide homeless-tailored wrap-around services and support once participants were housed. Key respondent interviews consistently cited the shift to housing first, the engagement with community partners, and use of real-time data as critical.

**Conclusions and Relevance:**

In this cohort study of the federal Ending Veteran Homelessness initiative, after program implementation, there was a substantially greater decrease in homelessness among veterans than in the general population. These findings suggest an important role for health systems in addressing complex social determinants of health. While some conditions unique to the VHA facilitated the change in homelessness, lessons learned here are applicable to other health systems.

## Key Points

**Question:**

What were the trends in homelessness among US veterans after implementation a Veterans Health Administration program to rehouse veterans experiencing homelessness?

**Findings:**

In this cohort study using a mixed-methods analysis of 13 years of program data, during implementation of the Ending Veteran Homelessness initiative, there was a 55.3% decrease in homelessness among veterans compared with an 8.6% decrease for the general population.

**Meaning:**

These findings suggest that health systems can play an important role in addressing community homelessness.

## Teaser

This cohort study compares changes in homelessness among US veterans with those in the general population after implementation of the Veterans Health Administration’s Ending Veteran Homelessness initiative.

## Introduction

Homelessness is a persistent problem in the US despite long-standing federal, state, and community efforts to address this need. While much of the focus on people who are unhoused centers around addiction, mental illnesses, physical disabilities, and chronic diseases that may be associated with individual homelessness,^1^ structural factors—the lack of affordable housing, livable wages, or access to services—play an equally important role.^2^

In 2010, the US Interagency Council on Homelessness, representing 19 federal agencies, released Opening Doors: Federal Strategic Plan to Prevent and End Homelessness.^3^ One of the hallmarks of their plan was the adoption of a systems approach, including the adoption of “housing first” as a policy as opposed to a preferred program model. Housing first emphasizes low-barrier, rapid, and streamlined access to permanent supportive housing. This contrasts with the historic approach of requiring unhoused people to meet threshold criteria (eg, sobriety, employment) prior to housing being made available. Supportive services, including substance use services, mental health care, and job training, among others, are then provided and more easily accessed once participants are housed. Other elements of the federal plan called for increased collaboration to leverage and integrate resources of mainstream systems for housing, employment, education, health care, and income supports as well as using data to measure and improve system and program performance.^3^

**Figure 1.**
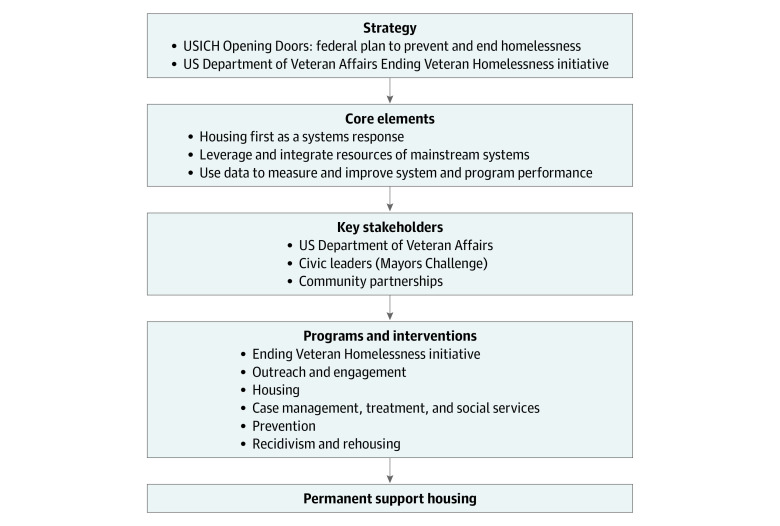
Ending Veteran Homelessness Initiative Strategic Approach USICH, US Interagency Council on Homelessness.

The US Department of Veterans Affairs (VA), as one of the participating federal agencies, actively implemented this plan with the goal of eliminating veteran homelessness by 2015.^4^ The Ending Veteran Homelessness initiative built on and expanded VA homeless programming to reflect a continuum of wrap-around services that supported placement and retention in permanent supportive housing. These included outreach and engagement services; homelessness prevention; emergency and transitional housing; the US Housing and Urban Development–Veterans Administration Supportive Housing program (HUD-VASH), which links HUD-provided Section 8 housing vouchers with VA-supported case management services and expanded and tailored physical, mental, and substance use disorders care; and access to additional social determinants of health interventions (food and income security, justice programming) (Figure 1). Key strategies of the Ending Veteran Homelessness initiative were the adoption of housing first as an agency policy and emphasizing partnerships with other federal agencies, state and local governments, and community agencies as operational partners.

In this report, we present data from a mixed-methods analysis to better understand the VA’s relative success in addressing the social determinants of housing security and reducing veteran homelessness. While the impact of the substantial financial support of this effort cannot be overstated, the specific role of policy changes, programming, community engagement, and the mobilization of an integrated health system to address social determinants of health needs are discussed.

## Methods

We conducted a cohort study using a mixed-methods analysis looking at 16 years of data (3 baseline years and 13 programmatic years) tracking housing placement, community grants, and PIT data that we contextualized to interview responses from VA leadership involved in implementing the Ending Veteran Homelessness initiative. We followed the Strengthening the Reporting of Observational Studies in Epidemiology (STROBE) reporting guideline when applicable for the annual PIT count assessments and veteran HUD-VASH housing placements during the course of the study period and the Consolidated Criteria for Reporting Qualitative Research (COREQ) guideline for qualitative research in presenting interview data. The data were collected for operational purposes and deemed as an operational evaluation under VA Handbook 1200.51.^5^ Use of deidentified PIT count and HUD-VASH data did not require informed consent; interviewees were informed of the intent of the project and provided written consent.

### Cohort

The cohort for this study represents operational data collected as part of the Ending Veteran Homelessness initiative. The longitudinal operational data of veterans experiencing homelessness comes from several sources, including HUD’s PIT annual incident count of general population and veteran-specific homelessness and VA operational data collected as part of this initiative. The initiative was conceived and preparatory steps were developed (including expanding existing programs and launching new programs) beginning in 2009. The baseline years are from January 1, 2007, to December 31, 2009, and action years are from January 1, 2010, to December 31, 2022.

### Data Elements

#### PIT Estimates

The PIT is an annual head count of persons who are unsheltered or living in an emergency shelter or in transitional housing on a given night in January of each year, so that year’s data are predominantly affected by the previous year’s program activities. The count is conducted by volunteers within each community and is sponsored by HUD, with deidentified data with limited demographic features reported for each continuum of care community in the US. A continuum of care is defined by HUD as “a regional or local planning body that coordinates housing and services funding for homeless families and individuals.”^6^ In the 2022 data, there were 388 continuum of care communities that reported data. The PIT count has been debated intensely, but it provides the best available national data on homelessness. Data on annualized national homelessness and veteran-specific homelessness counts are presented. It is not possible to track specific individuals across multiple PIT counts due to limits in the data collection and reporting process.

#### Housing Vouchers, Community Grants, and Housing Outcomes

We focused on 2 programs: one representative of a permanent housing approach (HUD-VASH) and the other a prevention intervention (Supportive Services for Veterans and Families [SSVF]). Both also represented a significant portion of the overall budgetary outlay for this initiative. From publicly available sources, specifically the federal budget, congressional testimony, and program office data, we present annualized data on the number of HUD-VASH vouchers and SSVF community grants awarded. We also present the overall number of veterans engaged in VA programming who were housed each year. In context, there are currently 2.07 million people living in Section 8 project housing and 5.23 million people using a Housing Choice voucher.^7^ However, the waitlist for those programs historically exceeds supply.

### Interviews

We conducted 8 semistructured interviews with Veterans Health Administration (VHA) Homeless Program Office leaders who were key figures during the implementation of the Ending Veteran Homelessness initiative. There were no nonparticipants. The interviews were methodologically oriented to a grounded theory of the underpinnings of the Ending Homeless Veteran initiative based on formative discussions with key leaders who were in place at the time of the initiative’s inception. These themes were further examined through purposive sampling of section and program leads responsible for operationalizing the initiative. The interviews focused on 3 thematic areas: (1) strategic changes, including new structures and programs needed within the VHA for this initiative; (2) policy changes that occurred; and (3) obstacles and challenges when implementing these policies along with mitigating steps. Interviews were conducted over email exchanges and telephone interviews with one of us (T.P.O.) who did not have any current supervisory relationship with any of the respondents.

### Statistical Analysis

In our analysis, we correlated the PIT data for the general population and veterans with programmatic data to describe differential effects of the Ending Veterans Homelessness initiative in the veteran cohort. We mapped those findings to HUD-VASH and SSVF program data and qualitative findings to support relational inferences of the noted trends. While direct causality could not be ascertained, there was value in our analytic approach to support our construct and conclusions.

To determine the 95% CIs for the annual PIT counts, we used the formula from the normal approximation, which allows for the use of population proportions to find an approximate distribution when the sample does not have the true population distribution. This approach was used to address data collection limitations inherent to the PIT. Standard errors were calculated using the sqrt[(P × [1 – P])]/n formula for proportions, while 95% CIs were calculated with X ± gnorm(0.975) × SE. Statistical significance (*P* < .05) among proportions of population subgroups in the general population and unhoused veterans in the 2022 and 2015 PIT counts was determined using 2-sample testing (Stata software, version 8 [StataCorp LLC]).

## Results

### Demographics

Limited demographic data on deidentified individuals were captured at each annual PIT count. In 2022, 33 129 veterans were identified; 88.7% were men; 10.4%, women; and 0.9% transgender, not singularly male or female, or gender questioning. In terms of race, 3.1% were American Indian or Alaska Native; 1.2%, Asian; 30.9%, Black; 1.3%, Native Hawaiian or Other Pacific Islander; 58.4%, White; and 5.1%, multiracial. A total of 12.2% were of Hispanic ethnicity. Compared with the general population of unhoused persons identified in 2022, more veterans were men (88.7% vs 60.6%; *P* < .001) and White (58.4% vs 50.0%; *P* < .001), and comparable proportions were unsheltered (40.9% vs 40.1%; *P* = .004). The 2022 veteran demographic data were consistent with the veteran PIT data collected in 2015 except that fewer veterans were unsheltered in 2015 (34.0% vs 40.9%; *P* < .001) (eTable in Supplement 1).

### Annual PIT Count

**Figure 2.**
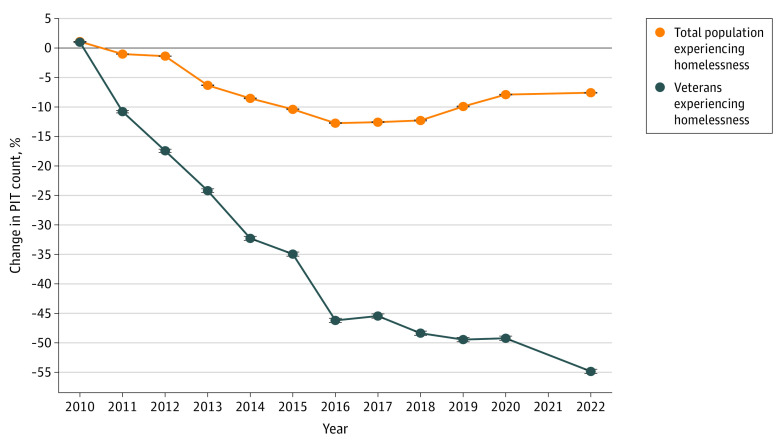
Change in Point-in-Time (PIT) Count From 2010 to 2022

Figure 2 shows the PIT count for veterans from 2007 to 2022. The PIT count for veterans increased substantially from 60 998 in 2007 to 74 087 in 2010 before steadily dropping to 33 129 in 2022, a 55.3% decline from the 2010 peak. There was also a decline in the proportion of unhoused veterans compared with the overall population of unhoused persons in the PIT count, from 11.6% in 2010 to 5.6% in 2022. From 2016 to 2022, there was an increase in the PIT count in the general population, from 549 928 in 2016 to 582 462 in 2022, with most of that increase occurring between 2018 and 2020. In contrast, aside from a small increase from 2016 to 2017, there was a steady decline in veteran homelessness during that time, from 39 471 in 2016 to 33 129 in 2022. Because the data are deidentified, it was not possible to ascertain how many veterans were recounted in subsequent PIT counts or lost to follow-up.

### Program Development and Trends

Table 1 shows the growth of the HUD-VASH program, measured by the cumulative number of Section 8 vouchers allocated by HUD to unhoused veterans. The program grew at a substantial rate initially before stabilizing between 2% and 5% between 2016 and 2020. These vouchers are awarded through local public housing authorities, with the most socioeconomically disadvantaged communities receiving proportionately more. Also shown in Table 1 is the number of community grants awarded each year by the SSVF program. From its inception in 2011 to 2022, the number of community agencies working with the SSVF program tripled from 85 to 261. In 2022, the program served 102 306 veterans and their families.

**Table 1.** HUD-VASH Allocated Vouchers, SSVF Community Contracts, and Veterans Placed in Permanent Housing From 2009 to 2022

	Study year													
	2009	2010	2011	2012	2013	2014	2015	2016	2017	2018	2019	2020	2021	2022
HUD-VASH vouchers allocated, No.	20 440	30 556	37 885	48 350	58 155	69 550	79 669	87 552	92 728	97 229	100 536	105 411	107 571	109 879
SSVF community grants, No.	NA	NA	85	151	319	NA	407^a^	380^b^	367^b^	288	252	271	266	261
Veterans placed in permanent housing, No.	NA	NA	NA	34 877	42 716	53 475	64 902	69 178	56 201	51 384	47 919	41 137	36 270	40 401

Abbreviations: HUD-VASH, US Housing and Urban Development–Veterans Administration Supportive Housing; NA, not applicable; SSVF, Supportive Services for Veterans and Families.

^a^ Combined 2014-2015 fiscal year awards.

^b^ Includes previous fiscal year.

Table 1 also includes the number of veterans placed in permanent housing each year. The number peaked in 2016 with 69 178 placed, coinciding with the largest number of SSVF community grants awarded in 2015 and 2016 and the largest increase in new HUD-VASH vouchers made available. In 2022, 40 401 veterans were placed in permanent housing, reversing a declining trend from that peak. These numbers do not include veterans who were prevented from becoming homeless or family members of unhoused veterans or of veterans at risk for homelessness who also benefited from these programs. Overall, 280 023 veterans were engaged in VHA homeless programs in 2022 and, cumulatively, an estimated 995 404 veterans and their families were either placed in permanent housing or prevented from becoming homeless between 2010 and 2022 (personal communication from the VHA Homeless Program Office; February 7, 2023).

### Qualitative Findings

A consistent theme among respondents was the synergy between adopting the housing first policy and the role of engaged partnerships in this initiative. This was shown in comments that housing first was not intended to be housing only and that the availability of wrap-around services provided by VHA medical centers needed to be actively incorporated into the community housing model. Another theme was the importance of real-time, actionable data (and data systems) that both increased accountability and the ability to pivot toward more effective strategies. Also noted in our semistructured interviews was the adoption of housing first and a focus on partnerships initially met with resistance from both community agencies and VA staff. There was community skepticism and concern about program requirements, data tracking, and accountability that came with these partnerships. The scope and context of the partnership was also not always clearly defined, leading to some confusion. Similarly, respondents noted that VA frontline staff voiced concerns about safety with the change in workspace and focus. Specific policies, requirements, and safeguards were needed to help alleviate this issue. Key comments from these interviews include the following 3 themes: (1) changes in strategy and philosophy toward homelessness and the role of VHA; (2) the effect of adopting a housing first policy, including areas and context when resistance was met; and (3) the role of operational partnerships and engagement with community agencies (Table 2).

**Table 2.** Themes and Key Comments From Interviews

Theme	Key comment
Changes in strategy and philosophy toward homelessness and the role of VHA	The shift from “managing homelessness” to “ending homelessness” was crucial, especially in shifting field staff’s focus from temporary solutions to permanent solutions. It helped encourage a stronger relationship between researchers, policymakers, and front-line providers.
Effect of adopting a “housing first” policy, including areas and context when resistance was met	Housing first served as an important counter to the “treatment first” model…which was a long-term obstacle to permanent housing for many veterans. It changed the entire approach to care for veterans experiencing homelessness in the VA’s continuum of care. Resistance...often came from a misunderstanding or misrepresentation of housing first that was really just more housing only…that completely ignored the often-intensive support and treatment that occurred with housing. A lot of people thought it could not work. Some continued using a high-barrier, service-first approach…seeing housing first as enabling and believing that housing should be “earned.”
Role of operational partnerships and engagement with community agencies	VA engaged in a more collaborative posture with communities…participating in case conferencing, data sharing, and coordinated outreach. VA medical centers were given guidance on engaging with community organizations, including participating in coordinating entry (of homeless veterans into the system) and in meeting specific milestones and benchmarks.

Abbreviations: VA, US Department of Veterans Affairs; VHA, Veterans Health Administration.

## Discussion

The PIT count is the current standard for quantifying community, population-specific, and national homelessness. While there are well-described limits to its interpretation,^8^ the PIT count provides an opportunity to assess and benchmark temporal changes among subgroups of people experiencing homelessness, in this case veterans, with the overall homeless population. These data showed a substantially greater decline in homelessness among veterans compared with the general population from 2010, when the Ending Veteran Homelessness program was being launched, through 2022, when it was brought to scale, ultimately reflecting a 55.3% decline in homelessness among veterans compared with an 8.6% decline in the general population.

The reason for this difference is likely multifactorial. The guidance and leadership of the US Interagency Council on Homelessness; the leadership commitment and accountability to outcomes by the Secretary of the VA that extended to program offices, regional networks, and medical center directors; the financial investment and support by the US Congress spanning 3 administrations; and the partnerships with other branches of government and community agencies were all likely impactful. These factors are consistent with previous work to better understand how factors outside clinical care affect population health and social determinants of health.^9^ The cross-sector alignment theory of change that resulted from that work, reported by Landers et al^10^ and Lanford et al,^11^ describes how health care, public health, and social services align and captures many of the elements noted in the Ending Veteran Homelessness initiative.

The interviews with project leaders underscored the importance of adopting housing first as a systemwide policy for housing placement. The housing first policy substantially changed who was being considered for housing placements, how permanent housing was considered an intervention, and how harm reduction and supportive services were incorporated. Equally important to the policy adoption was the VA’s investment in programs and services that allowed the model to work. The VA invested substantial resources in the development and expansion of case management support services, substance use disorder treatment, mental health services, and general medical care programming to veterans to provide the wrap-around care and supports needed once housed. Research examining fidelity to the housing first model identified this level of posthousing programming as a core domain element^12,13^ associated with longer periods of housing stability, reductions in inpatient and emergency department use, and increases in outpatient services.^14,15^ More broadly, the housing first policy reflected an implementation strategy of top-down policy changes coupled with grassroots investment in community-VHA partnerships. The combination was critical to ensuring the appropriate use of limited resources and community and health systems–level engagement of service providers.

The VHA also has certain advantages in building and implementing an initiative such as Ending Veteran Homelessness that other health systems may not experience. First, the moral imperative of meeting the needs of men and women who find themselves unhoused after they have served in the US military and whose homelessness is often a direct result of that military service^16^ represents a shared national priority. This has led to strong congressional oversight, monitoring, and support for this effort, which has been critical. Second, the VHA is the nation’s largest integrated health care system with a management infrastructure to support advanced population health care programming (including housing support services and community partnerships to provide housing), enhanced capabilities for tracking and monitoring, and a reimbursement model similar to capitation that is less reliant on episode-of-care charges and billing. Last, the VHA, as an integrated health care system, is well situated to support a housing first model with the supportive clinical, case management, and social services needed to make it work. This dynamic is not always present in private sector health systems, which as noted in the literature,^17,18^ can greatly challenge their ability to effectively intervene in a social determinant of health. However, these factors should not be construed to suggest that implementation of an initiative addressing homelessness is only possible in a federally funded, integrated health system. Academic health centers and community health systems have also successfully worked in this area. The framework of policy-driven processes that informs on-the-ground engagement and coordination of services is applicable broadly. It is also important to note the significant public health and societal impact of efforts to address homelessness that extends beyond the narrower scope of providing health services to the unhoused population that often defines the role of a health system.

### Limitations

There are several limitations and caveats to acknowledge when interpreting these findings. This was a retrospective analysis over an extended period looking at key variables likely influencing the relative accomplishments of the Ending Veteran Homelessness initiative. There may have been other factors, either internal or external to the VA, that might have been associated with the outcomes that were not identified or assessed. As noted previously, the PIT count has several well-described limitations that make determining missing data or loss to follow-up in a longitudinal assessment difficult. A formal correlation analysis of the different components of this initiative was beyond the scope of this study and the data available and should be considered in future research. As noted, the significant fiscal commitment to this effort, the strong congressional and Cabinet-level support, and the unique capacities of the VA to execute this project played important roles separate from any specific policy or program. Last, the qualitative data were limited to key leadership within the VHA Homeless Program Office when this initiative was launched. Their account of the project could have been impacted by recall bias; additional perspectives from other agencies, community groups, or veterans themselves were not collected.

## Conclusions

In this cohort study using a mixed-methods analysis of the federal Ending Veteran Homelessness initiative, after program implementation, there was a substantially greater decrease in homelessness among veterans than in the general population. This change occurred during a shift to housing first as agency policy to create low-barrier housing availability. The data demonstrated the role of a health system in addressing a complex social determinant of health. Along with leadership commitment and resources, working to a model of care, adopting key policies, developing partnerships, and making key investments in programming and supports were critical. While some of these advantages are unique to the VHA, there are other aspects and lessons learned that can be adopted by other health systems.

## Notes

Supplement 1. **eTable.** Demographics of the Homeless Population From 2015 and 2022 PIT Counts

Supplement 2.
Data Sharing Statement
