# Supplementary material for: Changes in Homelessness Among US Veterans After Implementation of the Ending Veteran Homelessness Initiative
Source: JAMA Netw Open. 2024 Jan 29;7(1):e2353778. doi: 10.1001/jamanetworkopen.2023.53778 (PMC10825721; doi:10.1001/jamanetworkopen.2023.53778)
Supplement: Supplement 1. — eTable. Demographics of the Homeless Population From 2015 and 2022 PIT Counts [file jamanetwopen-e2353778-s001.pdf]

Supplementary Online Content

O'Toole TP, Pape LM, Kane V, et al. Changes in homelessness among US veterans after implementation of the Ending Veteran Homelessness initiative. *JAMA Netw Open*. 2024;7(1):e2353778. doi:10.1001/jamanetworkopen.2023.53778

eTable. Demographics of the Homeless Population From 2015 and 2022 PIT Counts

This supplementary material has been provided by the authors to give readers additional information about their work.

© 2024 O'Toole TP et al. *JAMA Network Open*.

eTable. Demographics of the Homeless Population From 2015 and 2022 PIT Counts

**Demographics**		**2022**				**2015**			
		N General	%	N Veterans	%	N General	%	N Veterans	%
Overall Homeless		582,462		33,129		564,708		47,725	
Female		222,970	38.3%	3,440	10.4%	224,344	39.7%	4,338	9.1%
Male		352,836	60.6%	29,392	88.7%	339,075	60.0%	43,295	90.7%
Transgender		3,588	0.6%	141	0.4%	1,289	0.2%	92	0.2%
Not Singularly Female or Male*		2,481	0.4%	118	0.4%				
Gender Questioning*		609	0.1%	38	0.1%				
Non-Hispanic/Non-Latino		442,220	75.9%	29,086	87.8%	452,140	80.1%	42,577	89.2%
Hispanic/Latino		140,230	24.1%	4,043	12.2%	112,568	19.9%	5,148	10.8%
White		291,395	50.0%	19,355	58.4%	273,746	48.5%	27,101	56.8%
Black, African American, or African		217,366	37.3%	10,240	30.9%	227,937	40.4%	16,407	34.4%
Asian or Asian American		8,261	1.4%	404	1.2%	6,074	1.1%	351	0.7%
American Indian, Alaska Native, or Indigenous		19,618	3.4%	1,034	3.1%	15,136	2.7%	1,261	2.6%
Native Hawaiian or Other Pacific Islander		10,461	1.8%	417	1.3%	8,827	1.6%	369	0.8%
Multiple Races		35,383	6.1%	1,679	5.1%	32,988	5.8%	2,236	4.7%
Sheltered Homeless		348,630	59.9%	19,565	59.1%	391,440	69.3%	31,505	66.0%
Unsheltered Homeless		233,832	40.1%	13,564	40.9%	173,268	30.7%	16,220	34.0%

© 2024 O'Toole TP et al. *JAMA Network Open*.
