# Supplementary material for: Changes in Homelessness Among US Veterans After Implementation of the Ending Veteran Homelessness Initiative
Source: JAMA Netw Open. 2024 Jan 29;7(1):e2353778. doi: 10.1001/jamanetworkopen.2023.53778 (PMC10825721; doi:10.1001/jamanetworkopen.2023.53778)
Supplement: Supplement 2. — Data Sharing Statement [file jamanetwopen-e2353778-s002.pdf]

Data Sharing Statement

O'Toole. Changes in Homelessness Among US Veterans After Implementation of the Ending Veteran Homelessness Initiative. *JAMA Netw Open*. Published January 29, 2024. doi:10.1001/jamanetworkopen.2023.53778

Data
Data available: Yes
Data types: Deidentified participant data, Data (not involving human participants), Data dictionary
How to access data: thomas.otoole@v.gov
When available: With publication

Supporting Documents
Document types: None

Additional Information
Who can access the data: anyone
Types of analyses: any purpose
Mechanisms of data availability: data are publicly available in Congressional testimony and through HUD
